# Direct Purification of Digestate Using Polymeric Ultrafiltration Membranes: Influence of Materials on Filtration Behavior and Fouling Characteristics

**DOI:** 10.3390/membranes12090882

**Published:** 2022-09-13

**Authors:** Caide Yue, Yongxing Chen, Wanqin Zhang, Yunhao Zheng, Xuzhao Hu, Bin Shang

**Affiliations:** 1 Institute of Environment and Sustainable Development in Agriculture, Chinese Academy of Agricultural Sciences, Beijing 100081, China; 2 Key Laboratory of Energy Conservation and Waste Treatment of Agricultural Structures, Ministry of Agriculture and Rural Affairs, Beijing 100081, China

**Keywords:** digestate, ultrafiltration, polymeric materials, flux decline, biosorption

## Abstract

In-depth exploration of filtration behavior and fouling characteristics of polymeric ultrafiltration (UF) membranes can provide guidance for the selection of materials and the control of membrane fouling during the purification of digestate. In this study, four types of polymeric membranes, (polyethersulfone (PES), polysulfone (PS), polyvinylidene fluoride (PVDF), and polyacrylonitrile (PAN)), were employed to filter digestate from swine manure. The results showed that the viscosity of the digestate dropped from 45.0 ± 11.3 mPa·s to 18.0 ± 9.8 mPa·s, with an increase in temperature from 30.0 °C to 45.0 °C. The four membrane fluxes all increased by more than 30%, with the cross flow velocity increasing from 1.0 m s^−1^ to 2.0 m s^−1^. During the batch experiments, the flux maintenance abilities of the membranes were in the order: PAN > PS > PVDF > PES. There were no significant differences in the effects of membrane materials on the removal of COD, TN, and TP (*p* < 0.05). For UV_254_ removal efficiency, PS showed the highest efficiency (68.6%), while PVDF showed the lowest efficiency (63.4%). The major fouling type was irreversible hydraulic fouling, and the main elements of scaling were C, O, S, and Ca. *Pseudomonadales* were the dominant bacteria in the PS (26.2%) and in the PVDF (51.4%) fouling layers, while *Bacteroidales* were the dominant bacteria in the PES (26.8%) and in the PAN (14.7%) fouling layers. The flux recovery rates (FRRs) of the cleaning methods can be arranged as follows: NaClO > NaOH > Citric acid ≈ Tap water. After NaClO cleaning, the PVDF membrance showed the highest FRR (73.1%), and the PAN membrane showed the lowest FRR (30.1%).

## 1. Introduction

With the development of large-scale livestock and poultry breeding operations, a growing number of farms use the process of anaerobic digestion (AD) for manure treatment, which results in large amounts of digestate being discharged from AD plants. The digestate that originates from swine manure is rich in nitrogen (N), phosphorus (P), potassium (K), and organic matter (OM) and can be used for soil fertility improvement and crop growth promotion. However, the use of digestate on farmland has faced many challenges, especially the lack of sufficient land. Therefore, large amounts of digestate have accumulated in AD plants, resulting in serious environmental problems such as greenhouse gas emissions, water eutrophication, and antibiotic pollution [1,2,3]. Due to the imbalanced carbon/nitrogen ratio and effluent quality fluctuations, the performance of further biochemical treatments have shown low processing efficiency and unstable operations [4]. In addition, the available methods can not realize resource utilization of the digestate, and therefore, there is an urgent need to find a method that promotes resource utilization of digestate.

Nutrients such as N and K in digestate are mainly dissolved in the liquid fraction [5]. Increasing the nutrient concentration of digestate can effectively reduce transportation costs and promote more land access to digestate. Membrane technology can concentrate nutrients without changing the characteristics of digestate, which is regarded as an effective method for the recovery of nutrients and water resources [6]. The main membrane technologies used in the treatment of digestate are microfiltration (MF), ultrafiltration (UF), nanofiltration (NF), and reverse osmosis (RO). MF and UF can be used to remove suspended solids, colloidal substances, and fine particles [7,8]; NF and RO can be used to concentrate the soluble nutrients [2,9]. Generally, membrane materials used in liquid separation can be divided into organic and ceramic membranes. Organic membranes are made from polymer materials, and their production cost is relatively low. However, serious membrane fouling, which is caused by adsorption and adhesion of pollutants on the membranes’ surfaces or pores, has been found to severely influence the performance of UF purification [10].

In terms of polymer UF membrane fouling control during manure or digestate purification, Konieczny et al. used polyvinylidene fluoride (PVDF) UF (100 and 50 kDa) and polyethersulfone (PES) UF (10 and 5 kDa) for pig slurry purification, and found that the two-step UF technology (PVDF 50 kDa–PES 5 kDa) was the most effective [11]. López et al. compared the purification effects of two different configuration membrane modules, i.e., external tubular and submerged hollow fiber, and found that the external tubular membrane module was the most selective during the filtration of digestate [12]. To mitigate membrane fouling, Eum applied a vortex generating membrane system to recover nutrients from animal manure and digestate [13]. Guo compared membrane cleaning effects using different commercial chemical agents, including a weak acid, detergent, and an oxidant, and found that a weak acid showed a minor contribution to flux recovery. A combination of different types of cleaning agents was required to obtain a better cleaning effect [7].

As mentioned above, previous research on membrane fouling control during digestate purification has mostly been conducted from the aspects of process combination, pretreatment, membrane module optimization, and chemical cleaning. As we all know, there are various kinds of UF membrane materials, including cellulose acetate (CA), regenerated cellulose (RC), PES, polysulfone (PS), PVDF, polyacrylonitrile (PAN), etc. [14,15]. UF membranes of different materials have different pore structures, surface electrical properties, and roughness, which can affect the separation process and membrane fouling characteristics [16,17]. However, currently, there is a lack of research on the relationships between UF membrane materials and pollutants in digestate, especially the structure and properties of the fouling layer.

In this study, four types of polymeric membranes (PES, PS, PVDF, and PAN), were employed to filter digestate from swine manure. The objectives of this study were to investigate the effects of (1) the operating parameters on membrane flux, (2) the membrane materials on purification effects and fouling characteristics, and (3) the cleaning agents on flux recovery rates. The purification effect, membrane flux change, membrane flux recovery, and the membrane fouling characteristics of digestate filtered using different UF membranes were systematically studied. The results of this study provide useful information on the filtration behavior and fouling characteristics of polymeric ultrafiltration (UF) membranes and could provide guidance on the selection of materials and the control of membrane fouling during digestate purification.

## 2. Materials and Methods

### 2.1. Digestate Pretreatment

The digestate was collected from a biogas plant located in the Shunyi District, Beijing, China. The up-flow anaerobic solid reactor (USR) process of thermophilic anaerobic fermentative was used to treat swine manure (TS 6~8%) in this plant. The collected digestate samples were settled for 24 h and passed through a 200-mesh sieve (size of 75 μm) (Table 1).

### 2.2. UF System and Separation Process

**Figure 1:**
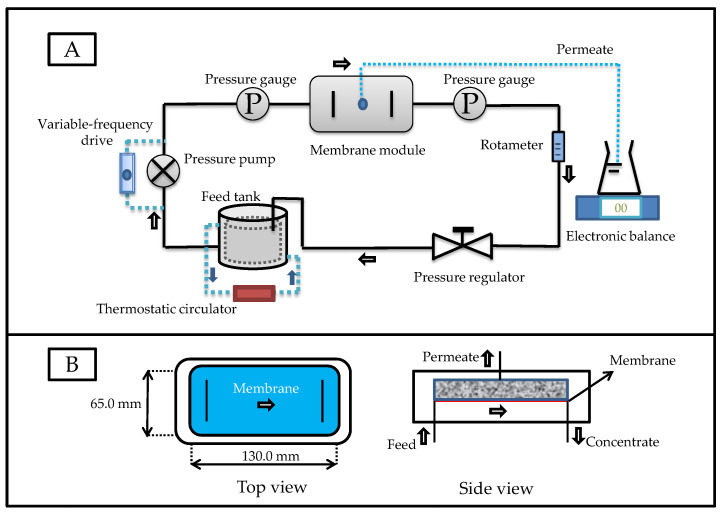
Flow charts of: (**A**) the flat-plate crossflow filtration device; (**B**) the membrane module.

A flat-plate crossflow filtration device was used for the digestate purification (Figure 1A), and this device has been described in our previously published study [18]. The main components and related parameters of the membrane separation system are listed in Table 2. The structure of the membrane module is shown in Figure 1B. The length, width, and height of the membrane module were 130.0 mm, 65.0 mm, and 1.3 mm, respectively, and the effective membrane area was 84.5 cm^2^. The polymeric membranes used in this experiment were provided by RisingSun Membrane Technology Co., Ltd (Beijing, China). After obtaining the membrane materials, we determined the characteristics of the commercial membranes, and the relevant parameters are shown in Table 3. Before purifying the digestate, the UF membranes were soaked in deionized water for 40 min, and the whole system operated with deionized water as the feedstock until flux remained stable. After opening the separation system, the digestate was filtered through a 200-mesh sieve and pumped into the membrane module. The permeate flow and operating pressure were controlled using a variable-frequency drive and pressure regulating value. Two pressure gauges were mounted at both sides of the membrane module for transmembrane pressure monitoring.

### 2.3. Experimental Design

In order to investigate the influence of the controllable variables on membrane flux, four types of polymeric membranes, i.e., PES, PS, PVDF, and PAN, were tested under different operating parameter values (cross flow velocity (CFV) values of 1.0, 1.5, and 2.0 m s^−1^; temperature values of 25.0, 30.0, 35.0, 40.0, and 45.0 °C), and each operating condition lasted for 5 min. The flux decline characteristics of the different polymeric membranes were detected under batch operating conditions (TMP, 0.3 bar; T, 25.0 °C; CFV, 1.5 m s^−1^) and the volume concentration factor was set to 3. The influent, concentrate, and permeate of each test batch were sampled, and the sampling volume was 250 mL. The samples were immediately stored under 4.0 °C conditions for later testing. After the batch experiments, scanning electron microscope-energy spectroscopy (SEM-EDS), atomic force microscopy (AFM), and attenuated total reflectance-Fourier transform infrared spectroscopy (ATR-FTIR) were used to diagnosis the morphology and composition of the fouled membrane. The microbial community of membrane fouling was analyzed using high-throughput 16S rDNA sequencing. Membrane cleaning was carried out at the end of the batch experiments; the volume of the cleaning solution was set to 3 L (duration of 30 min). The membrane flux recovery rate (FRR) of different chemical agents (i.e., sodium hydroxide (NaOH, 1 wt‰), citric acid (1 wt‰), and sodium hypochlorite (NaClO, 1 wt‰)) were compared. The detailed membrane cleaning procedures are described in our previous study [18].

### 2.4. Analysis Methods

The analysis methods and related instruments involved in this experiment are shown in Table 4. We chose pH, electric conductivity (EC), chemical oxygen demand (COD), ammonia nitrogen (NH_3_-N), total nitrogen (TN), potassium (K), total phosphorus (TP), total solid (TS), UV_254_, and total inorganic carbon (TIC) as the main indicators for the digestate purification effects. For the UV_254_ and excitation–emission matrix spectra (3D-EEM) measurements, the samples were filtered with 0.45 μm filter membrane and diluted 125 times to meet the measurement range. The characteristics of the virgin membranes and fouled membranes were analyzed by SEM-EDS, AFM, ATR-FTIR, and high-throughput 16S rDNA sequencing. The membrane samples used for the microbial community analysis were stored at −80 °C, and then transported to Shanghai Majorbio Bio-pharm Technology Co., Ltd. for analysis. DNA was extracted using a FastDNA^®^ Spin Kit for Soil (MP Biomedicals; Irvine, CA, USA), as per the manufacturer’s instructions. DNA purity and concentration were detected using a NanoDrop 2000 (Thermo Scientific; Waltham, MA USA) and DNA was electrophoresed on a 1% agarose gel for integrity detection. The quality of the PCR product was determined with 2% (*w*/*v*) agarose gel electrophoresis to ensure that no inhibition of the PCR took place [19]. The V4-V5 region of the extracted bacterial 16S rDNA genes was amplified by PCR reactions with primers 515F(5′-GTGCCAGCMGCCGCGG-3′) and 907R(5′-CCGTCAATTCMTTTRAGTTT-3′). The primers ITS1F (5′-CTTGGTCATTTAGAGGAAGTAA-3′) and ITS2R (5′-GCTGCGTTCTTCATCGATGC-3′) were employed to amplify the fungal ITS region. The archaeal genes were amplified using the primers 524F10extF(5′-TGYCAGCCGCCGCGGTAA-3′) and Arch958RmodR(5′-YCCGGCGTTGAVTCCAATT-3′). The amplifications were carried out using an ABI GeneAMP^®^ 9700 PCR thermocycler (ABI; Waltham, MA USA). The high-throughput sequencing on an Illumina MiSeq platform was conducted by Majorbio (Shanghai, China).

### 2.5. Membrane Performance

The membrane flux (*J*) was defined as the volume of liquid passing through per unit surface area per unit time, L m^−2^ h^−1^ [18]:

(1) $$J=\frac{V}{T\times A}$$

where *V* is the volume of the permeate (L), *A* is the membrane area (m^2^), and *T* is the filtration time (h).

CFV was the linear velocity of the flow tangential to the membrane surface (m s^−1^) [20]:

(2) $$\mathrm{CFV}=\frac{Q}{A^{\prime }}$$

where *Q* is the volumetric flow rate (m^3^ s^−1^) and *A*′ is the cross-sectional area of the flow channel (m^2^).

FRR was calculated by comparing the pure water flux before filtration and after cleaning (%) [18]:

(3) $$\mathrm{FRR}=\frac{J}{J_{0}}\times 100$$

where *J*_0_ is the pure water flux before filtration (L m^−2^ h^−1^) and *J* is the pure water flux after cleaning (L m^−2^ h^−1^).

### 2.6. Statistical Analysis

The raw data from the membrane separation equipment and water quality indicators were recorded and calculated using Microsoft Excel 2010. The Sigma plot software (Version 12.5, Systat Software, Inc; San Jose, CA, USA) and Origin 2018 were mainly used for plotting and data analysis. The statistical analysis was performed using IBM SPSS Statistics 20.0 for Windows (Armonk, NY, USA). One-way ANOVA was used to determine the significant differences (*p* < 0.05) among groups. The microbial diversity analysis was performed using the online platform of Majorbio Cloud Platform (www.majorbio.com (accessed on 28 July 2022)).

## 3. Results and Discussion

### 3.1. Effects of the Operating Parameters on Membrane Flux

**Figure 2:**
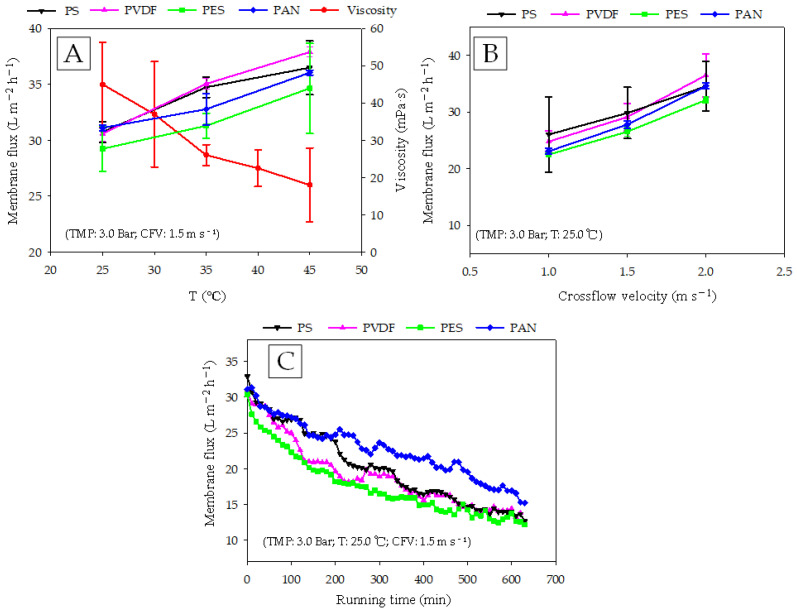
Effects of (**A**) temperature; (**B**) cross flow velocity (CFV) (**C**) running time, on the membrane fluxes using different polymeric ultrafiltration membranes.

As shown in Figure 2A, an increase in temperature improved membrane flux. The membrane fluxes of PVDF, PS, PES, and PAN were increased by 21.8%, 16.2%, 13.0%, and 15.3%, respectively, with an increase in temperature from 30.0 °C to 45.0 °C. The structure and thickness of a membrane and the feed property can affect the mass transfer rate of a membrane [14]. Generally, an increase in temperature results in a decrease in viscosity [21]. In this experiment, the viscosity of the digestate at different temperatures was measured, and the results showed that when the temperature increased from 30 °C to 45 °C, the viscosity dropped from 45.0 ± 11.3 mPa·s to 18.0 ± 9.8 mPa·s. The decrease in viscosity reduced the filtration resistance during the purification process. Although a higher temperature is beneficial to the separation process, it may have an irreversible effect on the structure of the membrane material. Stade studied the effects of temperature on the compaction of polymeric membranes, and found that the higher the temperature, the more serious the membrane compression [22]. In addition to the impact on the membrane structure, high temperatures can also change the properties of the feed characteristics, thereby, affecting the membrane fouling structure. Meabe’s study found that, as compared with mesophilic anMBR, thermophilic anMBR needed more attention to prevent membrane pore blockage caused by the smaller particles [23]. Hanvajanawong found that the addition of polyvinyl alcohol beads induced a structure change in organic foulants when treating high-load wastewater in a two-stage thermophilic anaerobic membrane bioreactor [24]. The results of this study provided a new idea for the prevention of membrane fouling encountered in our experiments.

It can be seen from Figure 2B that when the CFV increased from 1.0 m s^−1^ to 2.0 m s^−1^, the membrane fluxes of PVDF, PS, PES, and PAN increased from 24.8 ± 1.9, 26.0 ± 6.7, 22.5 ± 0.4, and 23.1 ± 0.5 L m^−2^ h^−1^ to 36.5 ± 3.7, 34.5 ± 4.4, 32.1 ± 0.6, and 34.6 ± 0.5 L m^−2^ h^−1^, respectively. The fluxes of the four membranes all increased by more than 30%, indicating that increasing the CFV is an effective way to improve separation efficiency. Guo used tubular UF membrane for the treatment of AD wastewater, and found that enhanced feed CFV resulted greater dislodging of gel-layer from the membrane surface and thinner gel-layer formation [7]. Saeki found that higher CFV suppressed the concentration polarization of nutrients on membrane surface by decreasing boundary film thickness and prevented bacterial growth [25]. The flow state in a membrane module depends on the temperature and CFV, as well as the flow channel structures, such as the turbulence generator, rotating module, and vibrating system [26,27]. These factors also have impacts on membrane fouling. Therefore, in the next step, we recommend a special flow channel design for digestate purification to alleviate the occurrence of membrane fouling.

Figure 2C presents the flux variation with the running time. In the first 200 min, the membrane fluxes of PVDF and PES decreased rapidly, while PAN and PS decreased relatively smoothly. After 630 min running, the membrane fluxes of PAN, PVDF, PS, and PES decreased by 51.1%, 60.3%, 61.7%, and 60.2%, respectively. From the perspective of the entire operating cycle, the flux maintenance abilities of the membranes were in the order: PAN > PS > PVDF > PES. The main factors that determine the difference in flux attenuation characteristics of UF membranes are membrane structure and membrane surface characteristics. Zhang investigated the adsorptive interaction between extracellular polymeric substances (EPS) and different UF membranes including PES, PAN, and PVDF, and found that the adsorptive fouling degrees of the three membranes were in the order: PAN < PVDF < PES. A much heavier irreversible fouling of PES UF membranes took place due to its relatively high roughness and hydrophobicity [28]. These results might explain the serious flux attenuations of the PES/PVDF membranes in our study, that is, the EPS contained in the digestate was more likely to be adsorb on the PES/PVDF membranes, causing membrane fouling. The PVDF membrane exhibited stronger hydrophobicity in the contact angle measurements. In general, hydrophobic membrane materials are more susceptible to fouling by organic contaminants.

### 3.2. Purification Effect of UF on Digestate

#### 3.2.1. Changes in Physicochemical Characteristics

Table 5 shows the characteristics of the influent, concentrate, and permeate. The main water quality indicators of the permeate of the four membranes did not show significant differences. After purification, the pH values of the permeates and concentrates did not change significantly as compared with the influent. The content of TS and COD in the permeates showed a significant decrease (*p* < 0.05); among them, the PVDF membrane had the highest COD removal efficiency (76.0%). The removal efficiencies of TP, NH_3_-N, TN, and K by UF were 43.9~47.2%, 20.5~27.3%, 20.3~32.8%, and 1.7~15.3%, respectively. The above results show that the UF materials have no obvious effects on the purification effects of the conventional indicators of digestate. The organic matters in the concentrates were higher than the influent, and it could be returned to the biogas plant to improve the gas production efficiency.

#### 3.2.2. Dissolved Organic Matter

**Figure 3:**
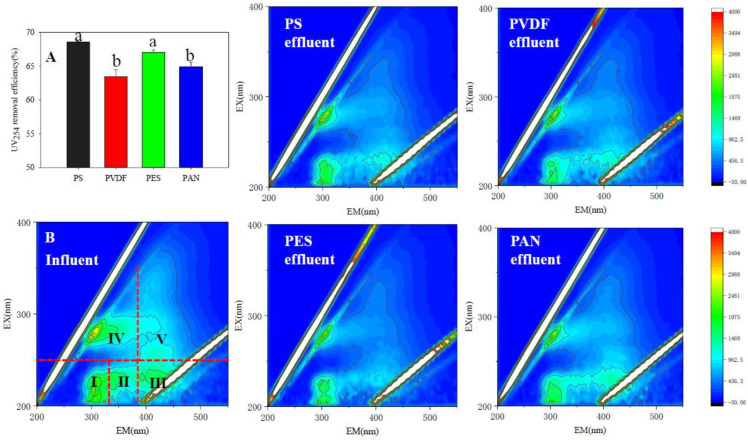
UV_254_ removal rates of UF membranes: (**A**) With different membrane characteristics (^a,b^—Values followed by the same letter are not significantly different at *p* < 0.05 ); (**B**) 3D-EEM profiles (Region I: simple aromatic proteins I; Region II: aromatic proteins II; Region III: fulvic acid-like substances; Region IV: soluble microbial by-product-like substances; Region V: humic acid-like substances).

As shown in Figure 3A, there are differences in the UV_254_ removal rates of the UF membranes. The PS and PES membranes have significantly higher UV_254_ removal rates than the PVDF and PAN membranes (*p* < 0.05). A 3D-EEM analysis was used to characterize the variation of fluorescent organic substances in the digestate, and the analysis results are shown in Figure 3B. The soluble organic fluorescent substances commonly recognized can be divided into five regions: simple aromatic proteins I (region I), aromatic proteins II (region II), fulvic acid-like substances (region III), soluble microbial by-product-like substances (SMBP, region IV), and humic acid-like substances (region V). Table 6 shows the integral standard volume of the fluorescence region before and after UF purification. The results showed that UF membranes of different materials have selective permeability to fluorescent substances in digestate. Among the four membranes, PAN allowed more aromatic proteins, aromatic proteins II, soluble microbial metabolites (SBMP), and humic acids to penetrate, while the PS membrane allowed more fulvic acid substances to pass through. This phenomenon may be related to the structure and hydrophobicity of membrane materials. Different membrane materials formed different membrane fouling layer structures in the purification process, resulting in differences in the filtration performance of organic matter.

### 3.3. Membrane Fouling Characteristics

#### 3.3.1. SEM-EDS

**Figure 4:**
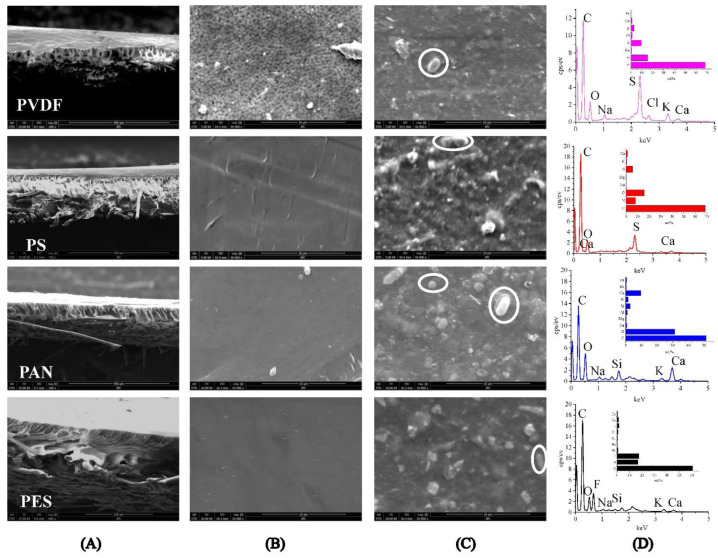
SEM images of: (**A**) Cross-sections of UF membranes (×800); (**B**) virgin membrane surface (×10,000); (**C**) fouled membrane surface (×10,000), microbes were identified by white circle; (**D**) EDS of pollutants.

As seen in Figure 4A, the cross-sectional structure of the PS, PAN, and PES membranes exhibit long fingerlike porous structures, while the PVDF membrane exhibits a cellular-like porous structure. After purification of the digestate, the UF membrane was covered by SS, colloidal substances, microbes, etc. (Figure 4C). The digestate contains EPS and SMP secreted by microorganism metabolism, and those metabolites can form agglomerates with microorganisms [29]. The agglomerates can adhere to the membrane surface and cause serious membrane fouling. Different membrane materials have different adsorption capacities for macromolecular substances produced by microbial metabolism, thus, exhibiting different anti-pollution properties [16]. The elemental analysis showed that the primary element contributing to membrane fouling were C (>50 wt%), O (>15 wt%), indicating organic matters were the major composition of membrane fouling (Figure 4D). The inorganic ions (S and Ca) in the fouling layer may be related to the adsorption of macromolecule organic matters [30]. From the perspective of membrane fouling control, we believe that organic matter in digestate can cause a serious composite fouling layer on the membrane surface. It is recommended to use flocculation, pre-filtration, etc. to reduce the content of organic matter in digestate, thereby, alleviating membrane fouling.

#### 3.3.2. Fouling Layer Characterization by AFM and ATR-FTIR

**Figure 5:**
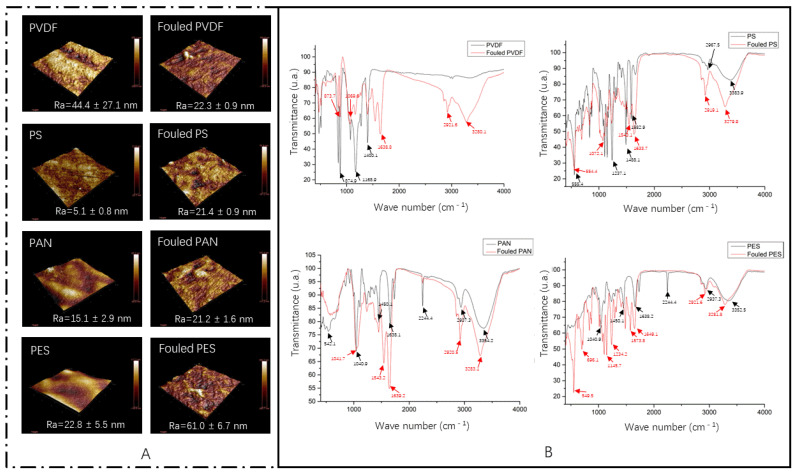
AFM images: (**A**) Clean membranes and fouled membranes. FTIR spectra: (**B**) Clean membranes and fouled membranes.

The AFM images of the virgin and fouled membranes are shown in Figure 5A. It can be seen that foulants are deposited on the membrane surface. The arithmetic mean roughness values (Ra) of the virgin and fouled membranes were also detected. Among the four virgin membranes, PVDF showed the highest Ra of 44.4 ± 27.1 nm and PS showed the lowest Ra of 5.1 ± 0.8 nm. After the digestate filtration, the Ra of the PVDF membrane decreased, while the Ra values of the other three membranes all increased. The ATR-FTIR spectra of the virgin and fouled UF membranes are shown in Figure 5B. The characteristic peaks of the virgin PVDF, PS, PAN, and PES membranes were 875 cm^−1^, 1168 cm^−1^, 1400 cm^−1^; 555 cm^−1^, 1237 cm^−1^, 1488 cm^−1^; 542 cm^−1^, 1041 cm^−1^, 1638 cm^−1^; and 1040 cm^−1^, 1450 cm^−1^, 2244 cm^−1^, 3352 cm^−1^, respectively. The appearance of these characteristic peaks mainly depends on the material properties of the membrane itself. After filtration, the fouling layer that developed on the polymeric UF membranes changed the characteristic peaks. The characteristic peaks of the fouled PVDF, PS, PAN, and PES membranes were 1638 cm^−1^, 2922 cm^−1^, 3280 cm^−1^; 1634 cm^−1^, 2919 cm^−1^, 3279 cm^−1^; 1639 cm^−1^, 2921 cm^−1^, 3283 cm^−1^; and 549 cm^−1^, 696 cm^−1^, 1145 cm^−1^, respectively. The peaks observed at 1634–1639 cm^−1^ are related to the vibration of amides I, showing the deposition of protein-like organic matter on the membrane surface. The peaks that appeared at 2919–2922 cm^−1^ are related to the aliphatic C-H stretching, showing the presence of humic substances [31]. Among the FTIR of the four membranes before and after fouling, the infrared spectra of the PVDF membranes showed the most obvious changes, indicating that the PVDF membrane suffered from the most complex organic matter adsorption.

#### 3.3.3. Microbial Community Analysis

**Figure 6:**
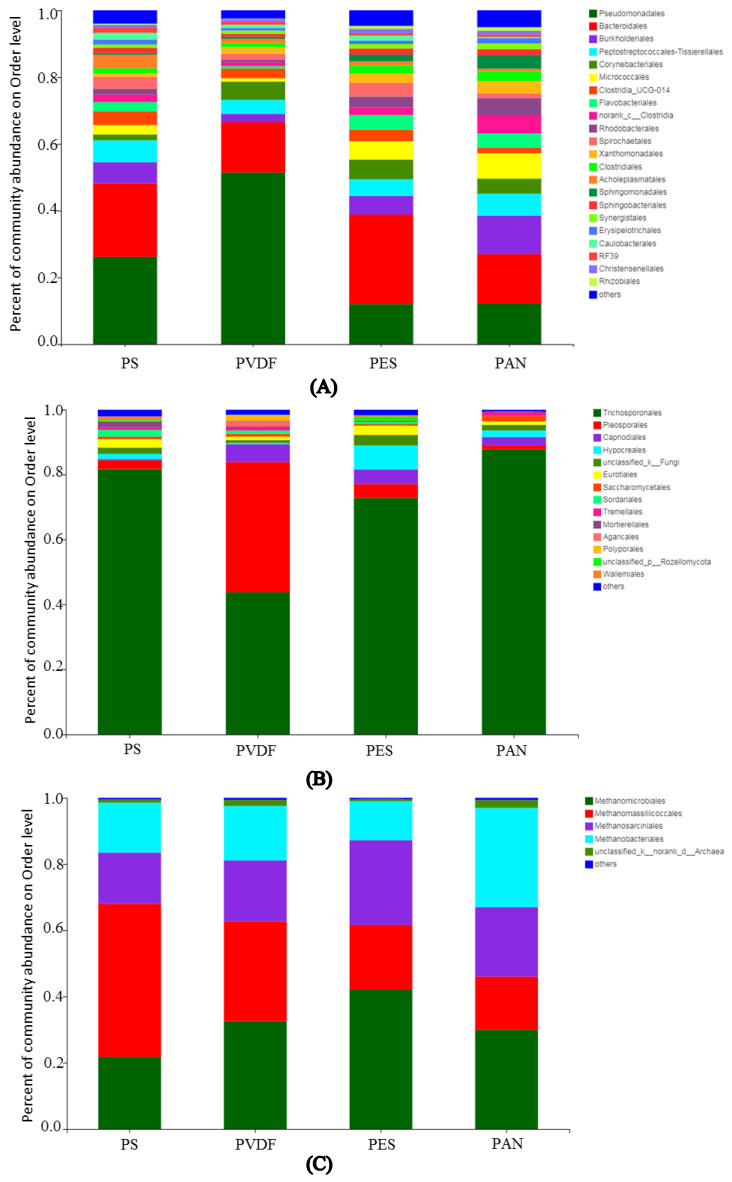
Microbial community abundances of: (**A**) bacteria; (**B**) fungus; (**C**) archaea on membrane fouling.

To identify the composition and structure of microorganisms in membrane fouling, the microbial community of membrane fouling was analyzed using the high-throughput 16S rDNA sequencing. As shown in Figure 6, more than 20 types of bacterial microorganisms were detected on the surface of the UF membrane at the phylum level. Among them, *Pseudomonadales* were the dominant bacteria in the fouling layers of the PS (26.2%) and PVDF (51.4%) membranes, while *Bacteroidales* were the dominant bacteria in the the fouling layers of the PES (26.8%) and PAN (14.7%) membranes. Other bacteria such as *Burkholderiales* and *Corynebacteriales* were the second most dominiant, only to the first two bacteria. *Trichosporonales* were the dominant fungal microorganisms in the four fouled membranes. *Pleosporales* fungi also accounted for a large proportion of the PVDF membrane fouling, with a relative abundance of 40.1%. *Methanogens* were the dominant archaea in anaerobic fermentation process. Therefore, the archaeal microorganisms detected in membrane contamination were mainly methanogenic Archaea. In the PES and PVDF membrane fouling, *Methanomicrobiales* were the dominant bacteria, with relative abundances of 41.9% and 32.6%, respectively. The dominant archaea in the PS membrane fouling were *Methanomassiliicoccales*, with a relative abundance of 46.2%; the dominant archaea in the PAN membrane fouling were *Methanobacteriales*, with a relative abundance of 29.9%. The differences in the composition of fouling microorganisms among different membrane materials are mainly affected by the hydrophilic–hydrophobic properties of membrane surfaces, membrane surface charge, and membrane surface roughness [32]. Due to the extremely complicated mechanism of microbial contamination on a membrane surface caused by digestate, this study only examined the structure of the microbial community adsorbed on the membrane surface and did not thoroughly study the adsorption mechanism of membrane materials and microorganisms in the biogas slurry.

#### 3.3.4. Membrane Cleaning

**Figure 7:**
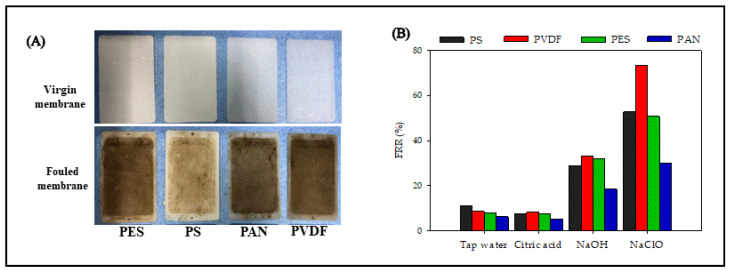
Cleaning effect of UF membranes with different materials: (**A**) digital pictures for the virgin and fouled membranes, (**B**) the membrane flux recovery rate of different chemical agents.

Figure 7A shows photographs of the virgin and fouled UF membranes. The fouled membranes formed a gel-layer on the membrane surface, and also deposited some organic matter in the membrane pores. The color of the filtered membrane gradually turned yellow. After washing with tap water, the FRR of the PS, PVDF, PES, and PAN membranes were only 11.1%, 8.6%, 7.9%, and 6.2%, respectively, indicating that the UF membrane fouling was dominated by hydraulic irreversible fouling (Figure 7B). A previous study has shown that citric acid could accelerate the dissolution of inorganic salts in membrane fouling [15]. However, the FRR of the four membranes after citric acid cleaning were less than 10%, which were similar to the cleaning effect obtained by tap water washing. The cleaning effects of NaOH and NaClO were better than that of tap water and citric acid. NaClO showed the best cleaning effect, and the FRRs of the four membranes could be arranged as follows: PVDF > PS > PES > PAN. The FRR of PAN after NaClO cleaning was still only 30.1%, indicating that the combination of PAN membrane and certain pollutant components caused serious chemical irreversible pollution. Bildyukevich studied the correlation between membrane materials and membrane fouling in skim milk ultrafiltration, and found that the high normalized dipole moment of the PAN membrane caused higher protein adsorption than other polymer membranes [14].

## 4. Conclusions

Four types of polymeric membranes, i.e., PES, PS, PVDF, and PAN, were employed to filter digestate from swine manure. The results showed the viscosity of digestate dropped from 45.0 ± 11.3 mPa·s to 18.0 ± 9.8 mPa·s, with an increase in temperature from 30.0 °C to 45.0 °C. The four membrane fluxes all increased by more than 30%, with CFV increasing from 1.0 m s^−1^ to 2.0 m s^−1^. During the batch experiments, the flux maintenance abilities of the membranes were in the order: PAN > PS > PVDF > PES. For UV_254_ removal efficiency, PS showed the highest efficiency (68.6%), while PVDF showed the lowest efficiency (63.4%). The main elements of scaling were C, O, S, and Ca. *Pseudomonadales* were the dominant bacteria in the PS (26.2%) and PVDF (51.4%) membrane fouling, while *Bacteroidales* were the dominant bacteria in the PES (26.8%) and PAN (14.7%) membrane fouling. The FRR of the cleaning methods can be arranged as follows: NaClO > NaOH > citric acid µ ≈ tap water. After NaClO cleaning, the PVDF membrane showed the highest FRR (73.1%), and the PAN membrane showed the lowest FRR (30.1%).

## Figures and Tables

**Table 1:** Digestate characteristics after filtration using a 200-mesh sieve.

Types	pH	EC/(ms cm^−1^)	TS/(mg L^−1^)	COD/(mg L^−1^)	TP/(mg L^−1^)	NH_3_-N/(mg L^−1^)	TN/(mg L^−1^)	K/(mg L^−1^)	TIC/(mg L^−1^)
Digestate	8.96 ± 0.13	9.2 ± 0.2	15.5 ± 0.7	12,275 ± 1025.3	185.9 ± 39.0	2035 ± 14.1	3200 ± 282.8	3799.5 ± 26.2	9552 ± 445.5

**Table 2:** Main components and related parameters of the membrane separation system.

Components	Number	Main Parameters	Manufacturer
Feed tank	1	Volume, 3 L	Xiamen Fumei Technology Co., Ltd; Xiamen, China
Volumetric flask	2	Volume, 500 mL	Sichuan Shubo (Group) Co., Ltd; Chengdu, China
Pressure gauge	2	Range, 0–10 bar	Yuyao Zhenxing Flowmeter Instrument Factory; Yuyao, China
Electronic balance	1	Max: 800 g; Accuracy class II	Shanghai Tianmei Balance Instrument Co., Ltd; Shanghai, China
Thermostatic circulator	1	Model, DTY-30B; range 20–50 ℃	Beijing Detianyou Technology Development Co., Ltd; Beijing, China
Three-phase induction motor	1	Type, MS 100LN-6B, 1.5 KW	LEUCO S.p.A; Reggio Emilia, Italy
Piston diaphragm pump	1	HAWK, Model NMT1520ESR	LEUCO S.p.A; Reggio Emilia, Italy
Membrane module and membrane	1	See Figure 1B	Xiamen Fumei Technology Co., Ltd; Xiamen, China RisingSun Membrane technology Co., Ltd; Beijing, China
Rotameter	1	Range, 0–20 LPM	Yuyao Zhenxing Flowmeter Instrument Factory; Yuyao, China

**Table 3:** Characteristics of commercial membranes used in this study.

Membrane Material	MWCO (Da)	Contact Angle (CA) (°)	Surface Roughness (Ra) (nm)	Pure Water Flux (L m^−2^ h^−1^) ^1^
PS	50,000	73.9 ± 2.1	5.1 ± 0.8	438.8 ± 90.8
PVDF	50,000	81.5 ± 1.9	44.4 ± 27.1	965.7 ± 127.9
PES	50,000	61.9 ± 1.2	22.8 ± 5.5	688.8 ± 35.1
PAN	50,000	54.4 ± 2.6	15.1 ± 2.9	576.6 ± 77.8

^1^ Test condition: transmembrane pressure (TMP) = 3.0 bar, T = 25.0 °C, CFV = 1.5 m s^−1^.

**Table 4:** Main tested parameters, instruments, and methods in this experiment.

Parameters	Instruments	Methods
Ph	Five Go F2, METTLER; Zurich, Switzerland	Electrode
EC	Five Go F3, METTLER; Zurich, Switzerland	Electrode
COD	DR 6000, HACH; Loveland, CO, USA	Potassium dichromate method
NH_3_-N	DR 6000, HACH; Loveland, CO, USA	Salicylic acid hypochlorite photometry method
TN	DR 6000, HACH; Loveland, CO, USA	The Chinese Standard (HJ 636-2012)
K	Atomic absorption spectrometry, ContrAA 700; Jena, Germany	The Chinese Standard (GB 11904-1989)
TP	Spectrumlab S22pc; Shanghai, China	The Chinese Standard (GB/T 11893-1989)
TS	Drying oven, Renggli TC-400, Salvin Lab; Rotjreuz, Switzerland	105 °C,
TIC	TOC analyzer, Elementar; Frankfurt, Germany	The Chinese Standard (GB/T 13193-1991)
CA	OCA15EC, Dataphysics Instruments GmbH; Filderstadt, Germany	Electron microscope, image capture
UV_254_	UV spectrophotometer, UV-2550, Shimadzu; Kyoto, Japan	254 nm UV light
3D-EEM	F-4700 fluorescence spectrophotometer, Hitachi; Tokyo, Japan	Excitation–emission matrix spectra
SEM-EDS	Hitachi S-4800, Hitachi; Tokyo, Japan	Electron microscopy and X-ray spectroscopy
AFM	Bruker Dimenson ICON; Billerica, MA, USA	-
ATR-FTIR	Thermo Scientific Nicolet iS5; Waltham, MA, USA	-

**Table 5:** Physicochemical characteristics of the influent, concentrates, and permeates.

Treatment	Types	pH	EC/(ms cm^−1^)	TS/(mg L^−1^)	COD/(mg L^−1^)	TP/(mg L^−1^)	NH_3_-N/(mg L^−1^)	TN/(mg L^−1^)	K/(mg L^−1^)
	Influent	8.96 ± 0.13 ^a^	9.2 ± 0.2 ^a^	15.5 ± 0.7 ^b^	12,275 ± 1025.3 ^b^	185.9 ± 39.0 ^a^	2035 ± 14.1 ^a^	3200 ± 282.8 ^a,b^	3799.5 ± 26.2 ^a^
PS	Concentrate	9.1 ± 0.1 ^a^	8.8 ± 0.1 ^a,b^	24.5 ± 0.7 ^a^	27,200 ± 424.3 ^a^	251.3 ± 7.2 ^a^	1895 ± 7.1 ^a^	3400 ± 0.01 ^a,b^	3614 ± 222.0 ^a^
	Permeate	9.2 ± 0.04 ^a^	8.2 ± 0.01 ^b^	8.0 ± 1.4 ^c^	3400 ± 282.8 ^c^	98 ± 7.2 ^b^	1520 ± 28.3 ^b,c^	2150 ± 70.7 ^c^	3298.5 ± 297.7 ^a^
PVDF	Concentrate	9.2 ± 0.03 ^a^	8.6 ± 0.1 ^a,b^	19.0 ± 4.2 ^a,b^	24,200 ± 565 ^a^	192.1 ± 10.1 ^a^	1780 ± 183.8 ^a,b^	3100 ± 141.4 ^a,b^	3194.5 ± 782.8 ^a^
	Permeate	9.3 ± 0.04 ^a^	8.3 ± 0.07 ^b^	8.5 ± 0.7 ^c^	2950 ± 353.6 ^c^	99.1 ± 0.07 ^b^	1480 ± 84.9 ^c^	2150 ± 212.1 ^c^	3366.5 ± 600.3 ^a^
PES	Concentrate	9.1 ± 0.08 ^a^	8.6 ± 0.06 ^a,b^	23.0 ± 4.2 ^a,b^	27,400 ± 367.9 ^a^	252.3 ± 34.6 ^a^	1930 ± 70.7 ^a^	3700 ± 70.7 ^a^	3217 ± 461 ^a^
	Permeate	9.2 ± 0.04 ^a^	8.2 ± 0.07 ^b^	9.0 ± 1.4 ^c^	4000 ± 141.4 ^c^	101.1 ± 8.7 ^b^	1580 ± 14.1 ^b,c^	2550 ± 70.7 ^b,c^	3731.5 ± 325.9 ^a^
PAN	Concentrate	9.1 ± 0.1 ^a^	8.3 ± 0.4 ^b^	20.5 ± 3.5 ^a,b^	23,350 ± 3181.9 ^a^	224.8 ± 36.1 ^a^	1780 ± 70.7 ^a,b^	3400 ± 282.8 ^a,b^	3608 ± 469.5 ^a^
	Permeate	9.3 ± 0.1 ^a^	8.0 ± 0.3 ^b^	9.0 ± 0.1 ^c^	3400 ± 989.9 ^c^	104.2 ± 1.5 ^b^	1615 ± 7.1 ^b,c^	2350 ± 212.1 ^b,c^	3217 ± 485.1 ^a^

^a,b,c^—Values within columns followed by the same letter are not significantly different at *p* < 0.05.

**Table 6:** The integral standard volume of the fluorescence region before and after ultrafiltration using different membrane materials.

Region	Organic	Ex (nm)	Em (nm)	Integral Standard Volume (au·nm^2^)				
				Influent	PS Effluent	PVDF Effluent	PES Effluent	PAN Effluent
I	Aromatic proteins I	200~250	280~330	136,468	97,768	97,866	96,755	105,871
II	Aromatic proteins II	200~250	330~380	116,915	72,806	76,214	61,272	82,627
III	Fulvic acid-like	200~250	380~550	475,054	472,246	422,871	386,558	447,355
IV	SMBP	250~340	280~380	547,659	43,2198	383,674	347,842	474,927
V	Humic acid-like	340~400	380~550	556,674	393,454	345,436	316,562	430,629

## Data Availability

Data are contained within the article.
